# Present status of prostate cancer diagnosis, limitations, challenges, and future endeavors

**DOI:** 10.1080/07853890.2026.2659984

**Published:** 2026-08-03

**Authors:** Kirti Hooda, Mary Chatterjee, Aditya Prakash Sharma, Naveen Aggarwal, Jadab Sharma

**Affiliations:** a Centre for Nanoscience and Nanotechnology, Panjab University, Chandigarh, India; b Department of Biotechnology Engineering, UIET, Panjab University, Chandigarh, India; c Department of Urology, Postgraduate Institute of Medical Education and Research, Chandigarh, India; d Department of Computer Science and Engineering, UIET, Panjab University, Chandigarh, India

**Keywords:** Prostate cancer, multiple biomarkers, liquid biopsy, imaging technologies, overdiagnosis, invasive, prostate-specific antigen

## Abstract

**Background:** Detection of prostate cancer (PC) using standard methods such as Digital Rectal Exam (DRE) or Prostate-Specific Antigen Test (PSA) is still very challenging due to many factors. Molecular Biomarker Testing, for example, the Prostate Health Index (PHI) and 4Kscore, have also helped improve the accuracy of PC diagnoses but these methods still face challenges with issues of cost, reproducibility, and clinical integration into practice.

**Methods:** In addition to traditional testing methods, the use of PSMA-directed imaging to guide biopsy procedures using Multiparametric Magnetic Resonance Imaging (mpMRI) allows for better localization and characterization of lesions. Researchers have begun to develop and continue to innovate Molecular & Imaging processes, called Liquid Biopsies, that are now being used to assess health risk factors associated with PC, by sampling macro-molecules from various biological liquids. Recently, the use of Artificial Intelligence (AI) and Machine Learning Models for improving the speed and accuracy of lesion detection and gland segmentation has significantly increased both precision and consistency in these areas.

**Results:** However, there is still great concern regarding overdiagnosis and lack of standardization associated with all types of molecular and imaging techniques currently available.

**Conclusion:** This review provides a comprehensive overview of the currently available diagnostic modalities for prostate cancer, including their weaknesses as well as gaps in clinical translation and standardization, which can assist with providing guidance for future development of diagnostic innovations..

## HIGHLIGHTS

- The number of new cases of prostate cancer in global incidence over the next two decades is expected to double worldwide. The need for improved diagnostic tools that are more precise and less invasive is becoming increasingly urgent due to this projected increase.
- Liquid biopsies (CTCs, cfDNA/ctDNA, ctRNA, and exosomes) provide an opportunity for less-invasive assessment for diagnosis, prognosis, selection of therapy, and follow-up, but there are still significant barriers to their routine use: 1) limited sensitivity of these markers in patients in the early stages of disease; and 2) the absence of standardized protocols or reference materials for performing liquid biopsies.
- Emerging urine and blood biomarker panels demonstrate utility in distinguishing PC from BPH and for risk stratification according to Gleason score, but most of these are preliminary and require additional validation before they can be integrated into routine patient care.
- PET scans that look for prostate-specific membrane antigen, MRI images, and PSA blood test results provide key information for accurate diagnosis of and treatment planning for men with prostate cancer. Current imaging methods of differentiating prostate cancer lesions are DWI and PI-RADS, each of which is evaluated for risk, and help physicians determine what treatment options are available to them. Hybrid PET/MRI imaging produces both anatomical and functional imaging modalities, providing information for physicians to make more accurate diagnoses and more collaborative decisions about treatment.
- Artificial Intelligence (AI) is being used in the diagnosis, detection of lesions, prediction of risk, and treatment decisions based on cancer images, pathology, history, and patient data, but issues surrounding the quality of data, interpretability of models, and generalizability of results continue to restrict full adoption of AI in standard care pathways for prostate cancer.

## Introduction

Most common form of cancer in males is prostate cancer (PC) which is also the second greatest cause of cancer death in men. According to the International Agency for Research on Cancer (IARC) GLOBOCAN cancer statistics for 2020, annually, 1,414,259 new PC cases and 375,304 new fatalities were reported by the World Health Organization (WHO), and for the next 20 years, the mortality rate from PC is predicted to roughly double, reaching 2.3 million cases [1]. According to a recent data on PC screening, the percentage of advanced PC cases at diagnosis has significantly decreased since PSA testing was introduced [2]. Currently, available PC screening methods are the PSA blood test, DRE (Digital Rectal Examination) test, prostate imaging techniques using TRUS, magnetic resonance imaging (MRI), multiparametric MRI (mpMRI), and prostate biopsy which yield insightful results.

Total PSA blood testing and Digital Rectal Examination (DRE) continue to be the most important population-based tools for prostate cancer screening, but they are usually used in conjunction with further imaging (TRUS, MRI, and mpMRI) if either of these tests is elevated [2,3]. Although imaging has improved early detection and staging of prostate cancer, there are the potential problems of false positivity and unnecessary invasive procedures due to their low specificity [4]. The risks include pain, infection, erectile dysfunction, and urinary incontinence.

The Prostate Health Index (PHI) was developed using PSA molecular forms. PHI provides a more accurate risk assessment because it considers the combined levels of both free and total PSA along with the presence of ProPSA – ‘subtype 2’. PHI has had limited acceptance in clinical practice to date because the cost of testing and the associated issues related to analytical stability which have hampered its widespread use [5]. The 4Kscore takes into consideration the four kallikrein proteins (including the isoforms of PSA) as well as age, a person’s family history of prostate cancer (PC), Digital Rectal Exam (DRE) findings and whether or not a biopsy has been performed. All these informations for an individual is correlated to arrive at an individual’s risk of developing high-grade PC. The ExoDx Prostate (EPI), which is a urine assay that looks for the presence of a series of molecules (ERG, PCA3, SPDEF), can identify individuals who may be at risk for high-grade PC, especially when their PSA levels are in the ‘grey zone’. Compared to total PSA alone, PHI, the 4Kscore and ExoDx have been shown to demonstrate improved specificity for clinically significant disease; however, additional large-scale studies are required to validate these findings in more commonly used populations [5–7].

Clinical tools like PHI, 4Kscore, and ExoDx help physicians identify patients who can safely delay or avoid immediate biopsy while maintaining good detection rates for high-grade prostate cancer [5–7]. These tests also help eliminate unnecessary biopsies and their potential complications, reducing some of the risks associated with PSA-based screening and allowing patients to take a more personalized approach to treatment decisions [4].

Liquid biopsy methods (e.g. circulating tumor cells, blood-based nucleic acid analysis, etc.) provide insights into tumor genomic landscapes and clonal evolution that augment standard PSA risk assessment/stratification, therapeutic selection, and disease surveillance utilizing PSA alone [8,9]. Incorporating artificial intelligence to analyze imaging/pathological features may improve the accuracy of detecting lesions, reduce observer variability, and aid in the clinical decision-making process. However, the establishment of reliable, universal models through rigorous clinical research and testing becomes necessary prior to integrating artificial intelligence into routine prostate cancer (PCa) screening and diagnostic protocols [10,11].

Highly targeted and properly positioned early-stage PCa screenings remain integral to yield positive treatment responses by detecting clinically significant disease in a timely fashion. However, each patient should be assessed individually regarding when to initiate screening based on factors such as: age, family history/previous health problems (co-morbidities), and individual patient preferences [1,2,4].

The early detection of PC has caused an increase in the number of men diagnosed with localized stage disease, which in turn has increased the number of 5-year survival rates to nearly 100%. As such a patient group presents with an ideal opportunity for early curative intervention [12].

Currently, the biggest obstacle is not simply the diagnosis of PC but the differentiation of indolent disease from aggressive disease. The use of prognostic biomarkers in conjunction with clinicopathologic characteristics are required to determine treatment intensity and thus avoid overtreatment or undertreatment [13,14]. The Prostate Health Index (PHI) and 4Kscore tests combine various biomarkers with PSA to assess the likelihood of finding a high-grade prostate cancer in patients who undergo a biopsy procedure, which allows clinicians to make more accurate predictions about which patients should not have a biopsy for an indolent cancer [15].

Oncotype DX Genomic Prostate Score measures the level of gene expression in the prostate tissue of low-risk patients to help determine whether they should be placed on active surveillance, thus decreasing the incidence of overtreatment while determining which patients require a treatment intervention for an aggressive form of prostate cancer [16].

Detection of the AR-V7 splice variant circulating in tumor cells is predictive of whether patients will fail therapy with newer hormonal agents such as enzalutamide. Therefore, this provides information on when to stop ineffective treatments and when to pursue chemotherapy in patients with castration-resistant prostate cancer [17].

New developments of genomic classifiers such as Decipher offer additional refinement beyond traditional risk classifications, and are currently being validated in prospective clinical trials (NRG GU-009 & GU-010) with the goal of tailoring treatment to meet the individual needs of the patient based on the results of early diagnosis and molecular profiling, and therefore providing optimal chances of long-term metastasis-free survival and overall survival [18,19].

The objective of the current review article is to provide a critical assessment of available diagnostic methods for detection of prostate cancer in addition to newly developed diagnostic technologies that will potentially be used in clinical settings. This review article will also highlight very recent advances in molecular biomarkers, imaging methods, as well as artificial intelligence-based techniques and assess how well these advances may translate to clinical practice.

## Recent developments in imaging techniques

Recent developments in medical imaging have completely changed the diagnostic environment and have provided strong tools essential for detecting tumors and giving important information about their size, location, and aggressiveness for more effective PC identification.

**Figure 1.**
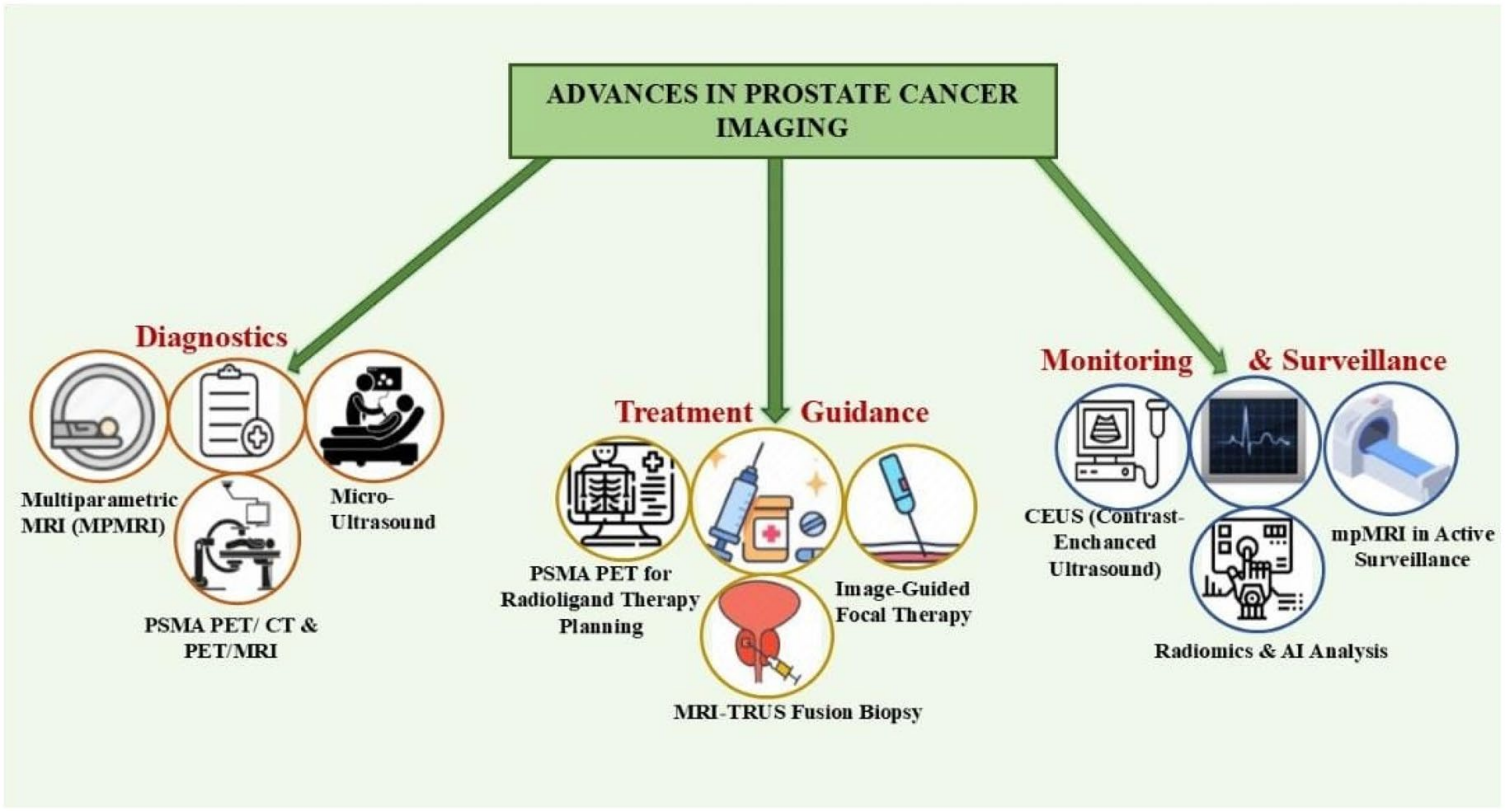
Imaging techniques available for detection of PC.

Figure 1 illustrates a schematic overview of recent prostate cancer imaging modalities, illustrating their roles in diagnosis, treatment planning and guidance, as well as monitoring and surveillance of disease. Prostate cancer (PC) is frequently diagnosed with transrectal ultrasonography (TRUS) because of its non-invasive, reasonably priced, and real-time imaging capabilities. TRUS imaging alone cannot differentiate prostate cancer from healthy tissue, so tissue samples from biopsy are still required for definitive diagnosis. The success of early diagnosis is decreased by TRUS-guided biopsies, which take a sample of the prostate without specifically aiming to identify cancer [20].

Combining MRI and ultrasound (US) is a viable substitute.

Although multiparametric MRI (mpMRI) lacks conventional standards for diagnosing particular lesions, it is frequently advised for individuals who have persistent PC suspicion despite previous negative biopsies. With fewer components and comparable accuracy without the dangers of contrast, biparametric MRI (bpMRI) is quickly emerging as a viable method for PC detection and biopsy guidance [21].

Diffusion-Weighted Imaging (DWI) is one of the most important techniques used in multiparametric prostate MRI. DWI provides non-invasive information about microstructure within the tissue and assists in identifying clinically relevant lesions, specifically those located in the peripheral zone, more effectively than other current imaging techniques. Improvements of MRI equipment have allowed for the use of ultrahigh b-value (≥2500s/mm^2^) DWI; however, in a prospective study of DWI performed by Bischoff and colleagues [22], they found that using ultrahigh b-values will decrease image quality, signal-to-noise ratio, and lesion visibility, as well as significantly increasing acquisition time when compared with using standard high b-value (∼1500s/mm^2^) DWI. Agreement with the PI-RADS system is high when using standard to moderately elevated b-values (1500–2500s/mm^2^), but it decreases significantly at ultrahigh b-values. This leads to reduced diagnostic confidence, and therefore, ultrahigh b-value DWI offers no added benefit and is not recommended for routine clinical use [22].

In order to standardize the acquisition, interpretation, and reporting of multiparametric MRI (mpMRI) to improve consistency and reduce variation between observers, the Prostate Imaging Reporting and Data System (PI-RADS) was created [23]. PI-RADS uses key sequences to create a standardized way to evaluate lesions based on a combination of T2-weighted images, diffusion-weighted images (DWI), and dynamic contrast-enhanced (DCE) images [24]. Currently, PI-RADS uses a scoring system that provides each lesion with a score of 1–5 reflecting the probability of clinically significant prostate cancer, with higher scores indicating a higher likelihood of aggressive disease. PI-RADS plays an important role in assessing risks and guiding biopsies by allowing targeted biopsies of suspected lesions and limiting unnecessary procedures for patients with low-risk results [25]. In addition to the current developments of PI-RADS, the most recent version, PI-RADS v2.1, has been developed in an attempt to continue improving the accuracy of diagnoses, decreasing the variability of inter-reader diagnosis, and increasing the detection of clinically significant prostate cancer, especially through the refined use of these major imaging techniques [26]. Although PI-RADS has been widely adopted within the field, it has its own limitations. These include variability between observers when evaluating the images, ambiguity related to intermediate (PI-RADS 3) lesions, and difficulties when evaluating lesions located in specific anatomical zones. Further refinements and the incorporation of advanced techniques, such as radiomics and artificial intelligence systems, are needed to enhance the use of PI-RADS in clinical practice [27].

Transperineal template biopsies systematically sample up to 20–50 cores from the prostate to diagnose CS-PCa at rates between 38 and 51%. Using this technique provides comprehensive sampling; however, it results in many biopsies taken from clinically insignificant cancers [28]. MRI-guided targeted biopsies, which take between 4 and 12 cores from areas identified on Multiparametric MRI as PIRADS three or higher, have comparable detection rates between 38 and 44% but have improved PPV (positive predictive value) and reduced risk of overdiagnosis [29]. The combination of templated and MRI-targeted biopsy approaches produces the highest detection rate of csPCa (62%) where each biopsy method upgrades an estimated 18-20% of previously undetected cancer missed by the alternative biopsy method [30]. The risk of infection from a transperineal biopsy (either templated or MRI) is less than 1% in contrast to 3-5% for a transrectal MRI-fusion biopsy, a guideline recommendation that favors the use of the transperineal biopsy method [28].

Since PSMA (Prostate-Specific Membrane Antigen) is highly expressed in prostate cancer (PC) and other malignancies, it is becoming more and more crucial for PC diagnosis and treatment [31]. Tumor detection is enhanced by PSMA-targeted imaging, especially when combined with PET/CT and PET/MRI, especially when there is biochemical recurrence and low PSA levels [32].

PC detection has significantly improved since the advent of 68Ga-PSMA PET/CT, which outperformed earlier tracers with varying accuracy (34%–88%), such as 11 C-choline, 18 F-fluoromethylcholine, and 11 C-acetate [33]. However, 68Ga’s high positron energy and brief half-life restrict its potential for therapeutic application. More recent tracers, like 18 F-DCFPyL and F-PSMA-1007, have higher resolution and longer half-lives, which makes them more useful, particularly for identifying PC recurrence in pelvic lesions [34,35].

More accurate PC staging is made possible by molecular imaging using PET/CT and PET/MRI, which also helps with treatment planning, early identification, disease monitoring, and therapy response assessment. From diagnosis to tracking metastases, this method promotes all-encompassing disease control [36]. PET/MRI hybrid imaging has become a new way to use imaging to diagnose prostate cancer. This type of hybrid method improves prostate cancer imaging appointment because it combines the soft-tissue resolution of MRI to the functional and chemical information got through PET imaging; therefore, this combined capability allows for better detection of the lesions, more accurate detection of the location of the lesions in relation to the rest of the body, and a better understanding of the biology (or cellular make up) of the cancer based on how it has behaved in the past. In particular, there have been many beneficial uses for PSMA-targeted PET/MRI imaging techniques, whether a fresh diagnosis in earlier stage or in the recurrence of prostate cancer. MR/PET imaging can directly guide treatment decisions for both cases [37]. Research has also recently confirmed that PET/MRI imaging can help determine response to treatment. A recent prospective study by [38] confirmed that PSMA expression increased in response to short-term androgen antagonist therapy and at the same time, apparent diffusion coefficient (ADC) values also increased. It showed a clear indication that the cancer was becoming less cellular. Additionally, dynamic PET/MRI parameters (vs. conventional standards) were more sensitive to the time from therapy to measure impacts of therapy. This perspective of both chemical and structural assessment of tissues by a multimodal imaging system (PET/MRI) adds to the understanding of how to determine the impacts of therapy on cancerous tissue [38].

The combined use of 11 C-choline PET/CT with MRI and proton MR spectroscopy has been particularly effective in patients with recurrent PC. Studies have demonstrated that 11 C-choline PET/CT is more effective than whole-body MRI with diffusion-weighted imaging (DWI) for detecting local recurrence and bone metastases [39]. However, 11 C-choline PET/CT has limitations in distinguishing benign prostate conditions, such as benign prostatic hyperplasia (BPH), from PC, reducing its overall diagnostic accuracy [40]. Similarly, 11 C- and 18 F-choline PET/MRI are effective in identifying and localizing PC within the prostate gland, lymph nodes, and metastatic sites. Phase 2 studies have evaluated these techniques against other imaging modalities and found them to be promising for localized detection [41].

Despite its effectiveness in detecting advanced PC, 11 C-choline PET/CT presents varying sensitivity and specificity across studies. Sensitivity rates range from 72% to 87%, while specificity rates fall between 62% and 84% [42]. These results indicate that while 11 C-choline PET/CT is useful for initial PC diagnosis, its precision may be influenced by factors such as tumor characteristics and patient demographics. Additionally, 11 C-choline PET/CT struggles with detecting small tumors and differentiating cancerous from benign tissue, particularly in cases involving BPH. Combining multiple metabolic indicators, such as SUVmax, SUVmean, and the prostate-to-muscle (P/M) ratio, has been found to improve diagnostic accuracy in early-stage PC [40,43].

Choline PET/CT scans have been reported to have a moderate overall detection rate (56-85%) for prostate cancer recurrence and bone metastases. On a per-lesion basis, choline PET/CT has demonstrated a higher sensitivity (84%) and specificity (93%) than that of conventional bone scan (BS) methods. The overall detection rate for PSMA PET/CT is about 78%, with the capabilities to detect the disease effectively at lower levels of prostate specific antigen (PSA) in the sample (54%, when compared with choline’s 27%, at PSA ≤ 1 ng/mL) [44,45]. Choline PET/CT has a much lower sensitivity (67-91%) and detection rate per patient, particularly for the detection of low-burden (complicated) recurrent lesions, than the higher performing PSMA PET/CT scans (89-95%). This means that choline PET/CT scans often miss low-burden nodal and bone cancer lesions because of their increased background uptake when patients have higher levels of PSA in their blood than is typically seen when patients present to doctors for prostate cancer treatment [46,47].

Compared with standard imaging like CT and MRI, Choline PET/CT has a higher specificity for detecting bone metastases (85-99%) as opposed to 75-82% for bone scintigraphy. However, it has a lower sensitivity per patient (choline: 85-99%/MRI: 91%/choline: 91%) when it comes to detecting bone metastases, (choline:85-99%) and also has superior detection of pelvic nodes and lesions in bone, while Choline PET/CT is able to pick up extra-skeletal disease better than MRI [44,48,49]. Several meta-analyses indicate that MRI has a better outcome than Choline PET/CT for determining the bone stage (higher area under curve) by using the patient as the basis of the comparison, although Choline PET/CT provides a good combination of both functional/information and anatomic data when evaluating the total body versus the limitations of CT’s structural view [44,50].

Despite these improvements, challenges remain in detecting early-stage PC using 11 C-choline PET/CT due to partial volume effects and activity spillover, which affect measurement accuracy. As such, 11 C-choline PET/CT may not be a reliable technique for identifying PC at early stages. Nevertheless, ongoing advancements in molecular imaging, particularly through PSMA-targeted imaging, continue to offer new avenues for improving PC diagnosis, staging, and management, providing hope for more effective treatments and better patient outcomes.mpMRI has a higher ability to detect clinically relevant malignancies than does TRUS (AUC 0.92) or PSMA-PET (AUC 0.81) and reduces the frequency of unnecessary needle biopsies, compared to PSA alone (reduce biopsy rate by RR 0.52). LFA (Low-cost Devices): provide fast results, low-cost point-of-care services, and are stable, however, they have lower validated specificity in large patient populations [51–53]. Combining mpMRI and LFAs can lead to an improved quality of care by allowing the use of mpMRI as a precision measurement of cancer staging and enabling a more efficient treatment pathway for patients through access to LFAs. For example, high-risk patients may be able to use mpMRI to direct where they will receive their biopsy, rather than through a traditional systematic approach [3].

## Liquid biopsy for PC

**Figure 2.**
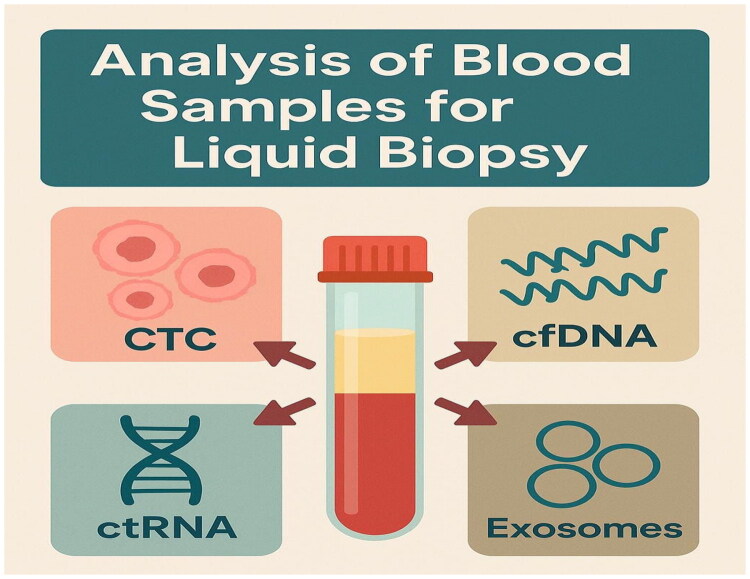
Analysis of blood components for liquid biopsy.

LB) can address key clinical areas such as diagnosis, prognosis, therapy selection, and treatment monitoring, offering a less invasive alternative to traditional biopsy techniques [8]. Figure 2 below represents the illustration of the major blood-derived analytes used in liquid biopsy, including circulating tumor cells (CTCs), cell-free DNA (cfDNA), circulating tumor RNA (ctRNA) and exosomes.

## Diagnostic use

Blood, urine, or semen can be tested using Liquid Biopsy (LB) tests to look for circulating tumor cells (CTCs), exosomes (EVs), circulating cell-free DNA (cfDNA), and circulating tumor RNA (ctRNA). The major advantage of LB tests over other testing methods is that they are not invasive, making them more attractive than traditional (tissue) biopsy for diagnosing prostate cancer (PC) [8,54,55].

CTCs can be a valuable biomarker for PC detection and early staging. Due to their specific mutation patterns and protein expression profiles, CTC genetic information can help verify that the patient has early stage PC, determine disease stage, and estimate disease aggressiveness; however, because of their relative rarity and a lack of standardisation, most CTC-based diagnostic approaches remain in the research stage [9,54, 56,57].

Circulating tumor DNA (ctDNA) is a form of cfDNA shed by necrotic and dying tumor cells. Although there is currently limited potential for using ctDNA for early-stage disease detection, this is mainly due to difficulties in detecting ctDNA from localized prostate cancer, where ctDNA represents a small proportion of total DNA. As a result, most diagnostic ctDNA approaches remain at the exploratory or early translational research stages [58–60].

A meta-analysis found cfDNA has a sensitivity and specificity of 0.81 and 0.83 respectively, and although this is similar to the sensitivity of PSA (0.93), it has a different specificity. The sensitivity of cfDNA is lower in early stages of PCa and because of cost, cfDNA can only be diagnostic in research settings or advanced PCa patients and cannot be used routinely to diagnose and detect early PCa [55,58, 61,62].

Although the non-invasive nature of cfDNA testing, combined with the comprehensive information about an individual’s entire genome, suggests that cfDNA is useful for early diagnosis of PCa and for determining treatment options, the very low levels of cfDNA found in very early tumour stages and the lack of standardised protocols to assess levels of cfDNA mean that cfDNA testing has not yet become a routine diagnostic option for PCa [55,58,63].ctRNA can detect PC by revealing gene expression patterns associated with PC, so they would be non-invasive tests compared to biopsy, but due to interference from other benign diseases like BPH, the best strategy is to use ctRNA in addition to imaging techniques and clinical data, so as of now ctRNA will still be primarily used in research for the diagnosis of PC [56,64,65].

Despite their current experimental condition, exosomes have potential as a new non-invasive diagnostic for prostate cancer (PC) due to their abundance & stability in biofluids, as well absorbing proteins, RNAs (ribonucleic acid),& lipids representative of PC tumour biology [66–68]. The lack of standardization and isolation methods are the major obstacles for widespread routine implementation of exosomal diagnostic potential in a clinical setting.

## Prognostic use

Circulating tumor cells (CTCs) provide information about tumor development and provide clinically significant prognostic information, as well as classification for different subtypes of mCRPC, therefore CTCs prognostication will be much closer to being implemented in clinical practice than early stage disease [9,54,69].

Liquid biopsy collection of CTCs provides an advantage over traditional biopsy collection in that CTC analysis considers the underlying tumor’s heterogeneous characteristics and can provide molecular profiles for prognostic purposes that reflect the tumor’s true characteristics. However, the variance of CTC isolation techniques and the methods used for analyzing CTCs creates variability in results between studies [56,70]. ctDNA and more extensive cfDNA profiles provide more accurate estimation of tumor burden compared to PSA alone, and also signal possible developing resistance to ADT sooner in metastatic disease, making both viable prognostic modalities primarily for advanced PC as opposed to localized PC [55,58–62,65].

By analysing cfDNA it is possible to obtain a comprehensive view of the genetic profile of metastatic prostate cancer (PC) and differentiate the more biologically aggressive forms of this disease. However, the widespread use of cfDNA for prognostication in the early stage of PC is hampered due to the poor sensitivity of cfDNA to early-stage tumours and insufficient standardisation of protocols for detection, resulting in most cfDNA prognostic signatures remaining at the research level [55,58,63].

By studying ctRNA it is possible to gain insight into the mechanisms associated with resistance to the androgen receptor (AR) and to the BRCA gene mutation in metastatic PC, allowing better prediction of both the treatment response and the course of the disease. Since ctRNA-based prognosis requires integration with imaging and clinical findings, ctRNA remains an emerging biomarker still under development for clinical practice [56,64,65].

Similarly, exosomes play important roles in supporting the growth of new blood vessels (angiogenesis), migration of tumour cells, and the spread of tumours (metastasis). They also have an extensive range of biomarkers that provide information about a cell’s capability of spreading and the risk it poses to other locations within the body.

The level of miR-375 and miR-1290 found in exosomes within the plasma of CRPC patients is linked to their overall survival, allowing for the adjustment of treatment regimens to reduce the chances of patients being undertreated [67]. An increase in circTFDP2 found in exosomes may correlate with higher Gleason scores and tumor progression; therefore, monitoring for these levels can help identify patients with aggressive disease and allow for increased monitoring of these patients. The presence of miR-141-3p in exosomes found in plasma may indicate the metastatic potential of that patient’s disease; therefore, identifying these levels may help physicians assess whether they will continue to monitor or intervene upon the late-stage disease [71]. Although barriers related to the technical limitations of current methods of collecting and analysing exosomes exist, the prognosis potential offered by exosomes is significant [66–68].

## Treatment monitoring

CTC-based liquid biopsy is advantageous for monitoring the treatment response and recurrence of diseases due to its non-invasive nature. It is a valid option for providing continuous molecular characterizations of the disease over time, allowing physicians to monitor real-time changes in disease processes during the treatment phase and following therapy in patients diagnosed at advanced and metastatic stages [70,72].

Real-time evaluations of the genetic abnormalities, protein expression profiles, and other biochemical markers associated with CTCs continue to provide insights regarding the evolving aggressiveness of tumors and therapeutic response to potential drugs [9,56,57].

However, limited access to rare cell samples and inconsistencies in the methods used to assess CTCs represent real obstacles to clinical use of CTC analyses. In the current scenario, CTC analysis remains primarily a diagnostic test for advanced prostate cancer, and therefore there is little or no utility in CTCs for the detection of localized prostate cancer. Advanced prostate cancer patients who have undergone a CTC analysis show a greater rate of successful treatment when compared to patients who did not have a CTC analysis [55,58–62].

While cfDNA is a non-invasive method for obtaining genomic information to monitor treatment effects and modify treatment in patients diagnosed with advanced disease, the low sensitivity of cfDNA for early stage disease and lack of standardisation are barriers to widespread clinical application [55,58,63].

Researchers have indicated that changes in the mechanism of androgen receptor resistance and other therapeutic targets for prostate cancer can be followed by assessing levels of ctRNA; however, there continues to be uncertainty about the clinical relevance of benign mimic ctRNA, as well as the need for confirmation through multiple modalities, which means that assessment of ctRNA remains primarily within the research setting [56,64,65].

Exosomes are a promising resource to track progression of cancer *via* invasion, metastasis, and treatment effects due to their stability and the high number of different molecules they carry. Currently, however, exosomes are predominately used in research due to the manpower required to isolate exosome samples, as well as the inconsistency of the yields of RNA obtained from exosome samples [66–68].

## Challenges and limitations in PC diagnosis

**Figure 3.**
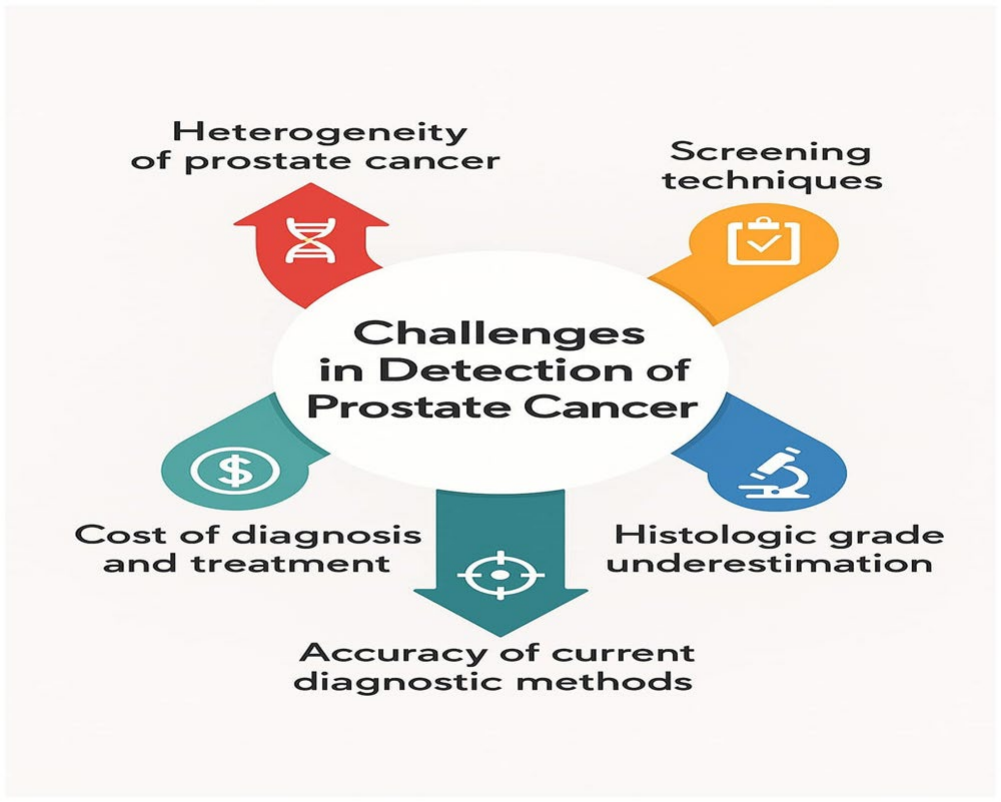
Challenges and limitations in the detection of PC.

The existing methods for prostate cancer (PC) screening, particularly PSA testing and digital rectal examination, do not provide an accurate method of distinguishing between aggressive and indolent prostate cancer [73]. Figure 3 below shows a schematic representation of key challenges in prostate cancer detection, including tumor heterogeneity, suboptimal screening techniques, histologic grade underestimation, limited diagnostic accuracy and the high cost of diagnosis and treatment.

The inability to differentiate between aggressive PC and indolent PC leads to overdiagnosing and overtreating PC that may never affect the individual [74]. In addition, needle biopsies frequently underestimate histologic grade and can result in both subjective interpretation of Gleason scores and missing high-grade foci in needle cores [75,76]. The way that needle biopsies are performed using traditional TRUS guidance may lead to varying amounts of tissue being sampled and thereby result in many instances of unnecessary biopsy, prostatectomy, and radiation treatment for individuals, especially for those that are classified as low risk (e.g. Black (African American) men), for whom no evidence exists regarding the specificity and negative predictive value of biomarkers to assess for prostate cancer risk [77,78].

Definitive therapy options for prostate cancer such as radical prostatectomy and radiotherapy result in severe long-term functional complications like urinary incontinence and erectile dysfunction, which can greatly affect the patient’s long-term quality of life. Radiotherapy carries the additional risk of causing bowel toxicity, radiation cystitis and secondary malignancies, which underscores the importance of correlating the potential therapeutic benefits with the limitations of these complications prior to administering treatment [79]. Additionally, androgen deprivation therapy (ADT), an integral part of the management of advanced prostate cancer, will cause patient to develop the metabolic syndrome and cardiovascular complications, as well as decrease bone density and increase the chance of fractures, especially with long term use of ADT [80].

PC diagnosis and treatment can create large financial costs to the public. In the United States, the financial burden of early-stage PC diagnosis and treatment can be as low as $10,612 and can rise to as high as $33,691 for advanced disease due to the fact that many of these cases were identified through inefficient, invasive methods of screening, creating additional costs [81]. Age, type of insurance coverage, and other household expenses will dictate how many patients receive screening [82]. In addition, while active surveillance methods (such as tissue-based gene expression testing) are available, the costs of these tests are still high and are also not very accurate when predicting the likelihood of Cancer progression [83]. Patients who are screened and monitored on a more frequent basis will save money per life-year saved, ($2,339–5,070), but many patients in diverse populations do not have equitable access to these screenings and monitoring tools due to gaps in validation for the high-risk populations [82,84,85].

The lack of standardised pathways for screening has been an impediment to implementing these advances into clinical practice, and the poor specificity of PSA for distinguishing clinically significant cancers has led to the risk of overdiagnosis (especially for Black men) [73,78,86]. The biological diversity within PC (i.e. intratumoral, intertumoral, and interpatient) limits the effectiveness of a ‘one-size-fits-all’ approach to therapy and increases the risk for both undertreatment and overtreatment [87,88]. There continues to be a challenge for machine learning models because they have been described as difficult to interpret; there is a need to conduct context-specific validation of the application of biomarkers (e.g. reduced biopsies and visibility of micrometastases); the emphasis of the next generation of personalised medicine must include the development of a standardised workflow and prospective clinical trials for these biomarker-specific applications [86].

## Uses of AI tools in diagnostics

PC diagnosis could be completely transformed by artificial intelligence (AI), which would increase precision, decrease inter-reviewer bias, and aid medical professionals in making decisions.

Table 1 summarizes the key applications of artificial intelligence in the diagnosis and management of prostate cancer.

**Table 1.** Role of artificial intelligence in enhancing diagnostic accuracy for prostate cancer.

AI application	Role in prostate cancer diagnostics	References
Precision & Bias Reduction	AI increases diagnostic precision, reduces inter-reviewer variability, and supports clinical decision-making.	[89]
Lesion Detection & Characterization	Aids in prostate gland segmentation and lesion assessment, improving tumor management.	[89]
Pattern Recognition	AI surpasses traditional methods by recognizing complex patterns in large datasets, improving accuracy and consistency in diagnosis.	[90]
mpMRI Interpretation	Enhances interpretation of multiparametric MRI, reducing inter-observer variability and bias.	[91]
Standardized Reporting	Facilitates consistent reporting and supports development of extensive diagnostic datasets and future AI models.	[91]
Predictive Modeling	Machine learning models (SVM, XGBoost, logistic regression, random forest, decision trees) significantly improve diagnostic accuracy (AUC) over PSA alone.	[92]
Biopsy Outcome Prediction	Integrates clinical variables (PSA, prostate volume, testosterone) to enhance prediction of biopsy outcomes and reduce unnecessary procedures.	[93]
PC vs. BPH Differentiation	Offers higher accuracy in distinguishing prostate cancer from benign prostatic hyperplasia.	[93]
Imaging Support	Supports MRI and ultrasound interpretation, aids in prostate gland segmentation and MRI-histopathology registration.	[10,94]
Histopathology Analysis	Translates histopathological images into actionable diagnostic insights, particularly when combined with mpMRI.	[10,94]

Prostate cancer clinical pathways will incorporate artificial intelligence through automation of mpMRI lesion segmentation/detection for prioritising targeted biopsy locations; standardisation of histopathology grading (Gleason scores) for biopsy review; and predictive risk score generation based on multi-modal (biopsy image plus prostate-specific antigen [PSA], age, stage) to support treatment selection (e.g. Androgen Deprivation Therapy [ADT] and radiation therapy) [95,96].

Radiomics is a relatively new way of analyzing images through a method of extracting quantitative properties from medical image data (or imaging data) that can be converted into record that can be utilized for clinical use. By using radiomics on multiparametric MRI scan data, itt is possible to detect prostate tumors, localize them, predict Gleason score, predict extracapsular extension, and predict biochemical recurrence, thereby it may improve the accuracy of risk assessment [97]. Recent studies have demonstrated that MRI radiomics models demonstrate very good diagnostic accuracy in predicting prostate cancer based on grade group and that several meta-analyses have found an area under the curve (AUC) of greater than 0.90 across multiple settings [98]. When combining MRI radiomics with clinically relevant imaging modalities such as PSMA PET/MRI, this predictive power is enhanced and improves the accuracy of detecting intraprostatic lesions and metastases [99].

An example of an AI tool is ArteraAI, which will be used to analyse post-biopsy slides along with clinical variables to determine the risk of metastasis and potential benefit from treatment, thereby assisting urologists and radiation oncologists in making decisions regarding active surveillance or treatment intensification using the current biopsy/MRI/trial pathways [100.).

Although AI has significantly improved the accuracy of mpMRI lesion detection (with some studies showing that inter-reader variability dropped from approximately 20%-30% to <10%), the generalisability of current studies is limited due to variability within the training sets and issues related to overfitting, black box transparency, and lack of multi-institutional prospective clinical trials [11,101].

The major barriers to implementation of AI applications include regulatory requirements (including FDA approval for Software as a Medical Device), the need for interoperability of picture archiving/communication systems (PACS) and electronic health records (EHRs) to provide seamless workflow integration, the need for external validation studies (including additional study cohorts to supplement the National Cancer Institute’s (NCI) Radiation Therapy Oncology Group’s [RTOG] and Phase I clinics) and the need for AI applications to be fully understood and trusted by physicians to allow the continued use of mpMRI-biopsy workflow [102,103].

Nonetheless, there are still issues with using AI for PC diagnostics. The requirement for massive, excellent datasets for AI model training is one of its main drawbacks. Overfitting, in which models perform well on training data but poorly on fresh data, can be caused by incomplete or biased data. Furthermore, AI models, particularly deep learning models, are sometimes viewed as ‘black boxes’, which makes it challenging to understand how they make decisions. In clinical contexts, this lack of transparency might impede confidence and adoption. Moreover, variations in the methods used by different institutions for data collecting, image processing, and model training may result in differences in the performance of AI models [101,104]. In recent review by Marletta et al. has elaborated on the barriers of AI tools and reported that significant progress is made towards addressing the clinical integration and validation challenges associated with using AI tools. Through validation studies these barriers can be overcomed and thereby AI tools may be utilized in everyday practice. Lack of external validation of AI tools, the differences in datasets across multiple healthcare institutions, and the necessity for performing multi-institutional, prospective trials are absolutely essential before approving AI tools for routine clinical use [105].

The ArteraAI platform uses multimodal artificial intelligence to analyze digitized prostate biopsy slides alongside clinical data (patient age, PSA levels, T-stage), identifying prognostic and predictive biomarkers for radiotherapy-treated localized prostate cancer patients [106,107].

The Model was trained on and validated across 5,700 patients participating in five phase III NRG/RTOG RCTs (NRG 9202, 9408, 9413, 9910, 0126). It predicts the likelihood of developing distant Metastatic Cancer, and the likelihood of deriving benefit from short-term androgen deprivation therapy in a defined subpopulation of patients; the hazard ratio of adverse event (Metastatic Cancer) for patients with biomarker-positive status is 0.34 (compared to those without) primarily in the context of favourable findings for intermediate-risk disease [108].

External validation studies demonstrate that this Model is effective. In the NRG/RTOG 9902 study population (high risk cohort), it outperformed NCCN Risk Group stratification criteria for predicting Distant Metastatic Cancer and Cancer-Specific Mortality. It was found to accurately predict Biochemical Recurrence Risk and Adverse Pathology in European Radical Prostatectomy cohorts. In patients with Oligometastatic Castration Sensitive Prostate Cancer, higher MMAI scores were found to correlate with decreased overall survival (HR 6.46) and with a shorter time to developing Castration Resistance [106,109,110]. This approach uses AI technologies to overcome the limitations of the Diagnostic Method by allowing personalised decisions regarding the use of ADT (alongside Radiotherapy), thus reducing Overtreatment Rates, while enhancing Patient Outcomes among the patient populations likely to benefit.

Standardizing processes, gathering data, and training models are crucial to maximizing AI’s potential in PC screening. AI has the potential to revolutionize PC diagnosis, management, and therapy with enhanced generalizability and transparency, particularly in situations with low resources and restricted access to professional care.

## Techniques for detection of PC biomarkers

Numerous new biomarkers have surfaced as viable options for PC early detection. These biomarkers include metabolites, mRNA, microRNA (miRNA), proteins, glycans, and tumor DNA. Investigating these biomarkers and developing detection methods could greatly improve PC diagnosis accuracy, especially when it comes to differentiating between benign disorders such as benign prostatic hyperplasia (BPH) and malignant malignancies.

Metabolomic analysis identifies key biomarkers of either blood or urine through NMR or mass spectrometry could help distinguish between prostate cancer (PCa) and benign prostatic hyperplasia (BPH), even when PSA testing alone does not provide enough information to make a definitive diagnosis. The differences in metabolism are in part due to the change in the way lipids and polyamines are metabolized with disease progression [111–113].

mRNA biomarker assays are capable of detecting PCA3, SPINK1, TMPRSS2-ERG fusion transcripts, and BMP6 in either urine or blood using quantitative polymerase chain reaction (qPCR) techniques and can provide improved diagnostic specificity and provide useful tools for both subtyping and monitoring disease progression independent of PSA analysis [111,114,115].

Circulating/urinary microRNA assays have been demonstrated to be stable, non-invasive biomarkers that can be used to differentiate between early-stage prostate cancer and benign prostatic hyperplasia and to indicate tumor size or burden in the body [114–116].

Multiplex panels, which combine multiple proteins together (PSA, PSMA, Bcl-2, Ki-67 and EZH2), aid in detecting prostate cancer (PCa) and risk stratifying patients based on their respective tumor aggressiveness levels when protein levels are measured using an immunoassay or an immunohistochemical approach [111,117].

Altered glycan expression and glycosylation profiles of glycoproteins (PSA and others) provides greater specificity for detection of prostate cancer because of the malignant transformation associated with prostate cancer compared to tissues from patients with benign prostatic hyperplasia (BPH), and these parameters can be distinguished by lectin-based assays and mass spectrometry [118,119].

Circulating tumour DNA (ctDNA) and methylated tumour DNA (mtDNA), obtained from plasma samples using highly sensitive PCR-based methods or next-generation sequencing techniques, are used for early detection of PCa and differentiation of benign from malignant disease, as well as for real-time monitoring of changes to the molecular make-up of the tumours (for example, the GSTP1 methylation status and DNA damage repair mutations) that impact prognosis and treatment options [114,115,120].

Clinical application readiness (FDA-clearance of the Progensa PCA3, and NCCN-recommended for the grey zone 4–10 ng/mL post-PSA) is higher for urine samples tested with the PCA3 & TMPRSS2:ERG tests than for nanoparticle-based experimental techniques, which have the potential to reduce the number of unnecessary biopsies (20 − 30%) and provide a non-invasive form of risk stratification (AUC between 0.70 and 0.82) [111]. The Progensa PCA3 test, using real-time quantitative polymerase chain reaction (RT-qPCR), tests post-digital rectal examination (DRE) urine and provides a PCA3 score of ≥ 25; where 55–72% specificity is achieved at 90% sensitivity (compared to Benign Prostate Hyperplasia (BPH)) [121]. The TMPRSS2:ERG fusion testing will identify approximately 47 − 50% of the ETS positive prostate cancers (PCs) and will provide approximately 94% negative predictive value (NPV) and a 28 − 35% reduction in biopsies when clinically integrated using RT-qPCR [121–124].

In the research phase are the following: Ionic liquid extraction technique (PSAG1 PSA ratio increase to 2–3 times; Pilot Study of 50 individuals; Bertok et al. 2021); NIR PSMA/Hsp90 Imaging Probes (3–5:1 Intraoperative Ratio) [31]; Zn sensors (LOD of 49.5 nanomolar; ZIP1 targeted) [125].; DIA-MS using serum SPP1/CPs demonstrated An area under the curve (AUC) of 0.89 for Gleason Scores of 7 or higher [126]; Urinary PDGF-BB ELISA testing (sensitivity 60% using the cut-off established, specificity 51% using cut-offs established [127], and MTNCA-MRI(ceramic) nanoparticles (4X accumulation); all lacking the Phase III/Approval stages. Diagnostic APT-MRI and PSA assisted with differentiating lesions in adjunct to conventional MRI [128].

Table 2 below shows the emerging techniques and analyte platforms for the detection of prostate cancer biomarkers, highlighting their diagnostic applications and supporting evidence from recent studies.

**Table 2.** Techniques for detection of PC biomarkers.

S No.	Technology used	Mechanism	References
1.	Urine Biomarkers detection by Ionic liquid-based aqueous biphasic systems (IL-based ABS) for pre-treatment of urine samples, followed by detection *via* size exclusion high-performance liquid chromatography (SE-HPLC).	The panel includes PCA3, TMPRSS2 (likely TMPRSS2:ERG fusion), and focuses on PSA isoforms (total PSA and bound PSA or bPSA), achieving 84% sensitivity and 45% specificity in differentiating BPH from prostate cancer.	[129,130,131,132]
2.	Near-infrared (NIR) Light-emitting Probes	NIR probes targeting PSMA and Hsp90 enable real-time intraoperative imaging, distinguishing slow-growing from aggressive prostate tumors.	[133,134,135]
3.	Zinc Transporter ZIP1 Protein and Zn^2+^ Sensor	Prostate cancer (PC) cells downregulate the zinc transporter ZIP1, a biomarker for early diagnosis, which is a highly selective and sensitive Zn²+ sensor based on the photo-induced electron transfer (PET) mechanism can target to enable early PC detection.	[136,137,138]
4.	Data-Independent Acquisition Mass Spectrometry (DIA-MS)	A DIA-MS based serum proteome analysis of BPH and PC patients identified differentially expressed proteins (DEPs) distinguishing aggressive from non-aggressive and early-stage PCa from BPH, highlighting potential biomarkers such as osteopontin (SPP1) and ceruloplasmin (CP).	[126,139]
5.	Blood-based mRNA expression profiling using transcriptomic analysis	Blood-based mRNA profiling enables PC screening, early diagnosis, and prognosis, with gene expression signatures showing predictive relevance for Gleason score classification.	[140,141]
6.	Quantitative measurement of urinary PDGF-BB using immunoassay-based techniques	Elevated urinary PDGF-BB levels indicate enhanced growth factor signaling in prostate tumorigenesis, improving diagnostic sensitivity by reflecting cancer-related changes in the tumor microenvironment and angiogenic activity.	[127]
7.	Multi-gene expression signature modeling derived from tumor transcriptomic data	A five-gene signature model predicting PC prognosis demonstrated high diagnostic and prognostic accuracy for metastasis and recurrence, outperforming traditional clinicopathological factors.	[142]
8.	Multi-Target Nanoparticles Contrast Agent (MTNCA)-MRI	Lenvatinib-loaded MTNCA effectively targets PC cells during MRI, showing promising pharmacodynamic and prognostic potential for early-stage PC diagnosis.	[143]
9.	Amide Proton Transfer (APT)-Weighted MRI	APT-weighted MRI detects endogenous mobile proteins and peptides *via* chemical exchange saturation transfer and, when combined with ADC values and PSA density, enhances differentiation between benign and malignant prostate lesions by capturing complementary metabolic and structural tissue characteristics.	[128]

## Conclusions and future directions

In conclusion, the landscape of identification and care of PC has changed due to recent advances in diagnosis. Improved precision and visualization are provided by medical imaging technologies, especially when MRI and ultrasound are combined, which improves diagnostic accuracy. One intriguing path for early diagnosis of PC is the hunt for new biomarkers, including proteins, DNA, mRNA, miRNA, and metabolites. Urine biomarkers with high sensitivity and specificity, like PCA3 and TMPRSS2:ERG, provide a non-invasive way to diagnose conditions. Ultimately, the application of liquid biopsy technologies and AI may significantly and fundamentally change monitoring of cancer progression and increase diagnostic accuracy. However, due to existing limitations around sensitivity, standardization, data access, interpretability, and regulatory approval, these technologies are still not widely adopted [69].

Some of the priority research needs for the future to address these limitations include creating standard workflows for liquid biopsy testing, conducting prospective multi-centre trials that evaluate the combination of imaging and biomarker data into superior combined algorithms, performing real-world cost-effectiveness studies of urine-based biomarkers, and validating AI tools with understandable outputs; future research efforts should focus on making head-to-head comparisons of these various methods, as well as conducting pragmatic implementation studies to reduce health inequities and improve overall outcomes across the board.

## Data Availability

Data sharing not applicable as no new data was generated.
